# 2228 The association of gastrointestinal symptoms and hypertension in persons living with HIV on HAART

**DOI:** 10.1017/cts.2018.305

**Published:** 2018-11-21

**Authors:** Kierra R. Butler, Faye Harrell, Wendy A. Henderson

**Affiliations:** National Institutes of Health

## Abstract

OBJECTIVES/SPECIFIC AIMS: The advent of Highly Active Antiretroviral Treatments (HAART) has allowed HIV-positive individuals to live longer in recent years. This has resulted in a higher incidence of mortalities occurring in these individuals due to cardiovascular pathologies, as opposed to deaths due to HIV. Even with long-term HAART, persons living with HIV (PLWHIV) still exhibit inflammation, which is associated with deleterious cardiovascular outcomes. PLWHIV on HAART have a higher prevalence of hypertension, which is associated with an increased risk of cardiovascular events. Moreover, chronic inflammation has been shown to be related to the translocation of microbes and endotoxins across the gastrointestinal tract. Such microbial translocation (MT) is increased in individuals with digestive disorders and their associated symptoms (e.g., diarrhea, abdominal pain, and nausea). This study aims to explore the pathologies common to both MT-induced inflammation and cardiovascular symptoms by examining the associations between gastrointestinal symptoms and hypertension in PLWHIV on HAART. METHODS/STUDY POPULATION: The sample included 351 PLWHIV on HAART. Pre-existing de-identified data were analyzed. Sample demographics included 56.98 % African Americans, 41.31% Caucasians, ages 20–66 years (mean age=43.65years), 21% female, 89% male, HIV viral load, CD4 counts. Self-reported data from the Symptom Co-Morbidity Questionnaire and Socio-demographic questionnaire were analyzed with SPSS v.24. RESULTS/ANTICIPATED RESULTS: In total, 86 PLWHIV (24.50%) stated that they have hypertension; 39 subjects (45.3%) reported having diarrhea, 30 subjects (34.8%) reported nausea, and 12 (13.9%) reported constipation and vomiting. Among ethnicities with hypertension and gastrointestinal symptoms, African Americans compared with Caucasians had a higher percentage of diarrhea (28% vs. 17%), nausea (21% vs. 11%), constipation (11% vs. 2%), and vomiting (8% vs. 5%). Women compared with men reported a higher percentage of nausea (28% vs. 24%) and constipation (8% vs. 6%). Men compared with women reported a higher percentage of diarrhea (38% vs. 7%) and vomiting (8% vs. 5%). DISCUSSION/SIGNIFICANCE OF IMPACT: These data support the need for targeted screening to include both blood pressure and associated gastrointestinal symptoms. Further studies supporting these results may assist practitioners to target treatments that may prevent cardiovascular comorbidities.

